# Association between increased visceral fat area and alterations in plasma fatty acid profile in overweight subjects: a cross-sectional study

**DOI:** 10.1186/s12944-017-0642-z

**Published:** 2017-12-19

**Authors:** Miso Kang, Ayoung Lee, Hye Jin Yoo, Minjoo Kim, Minkyung Kim, Dong Yeob Shin, Jong Ho Lee

**Affiliations:** 1 National Leading Research Laboratory of Clinical Nutrigenetics/Nutrigenomics, Department of Food and Nutrition, College of Human Ecology, Yonsei University, Seoul, 03722 South Korea; 2 Department of Food and Nutrition, Brain Korea 21 PLUS Project, College of Human Ecology, Yonsei University, Seoul, 03722 South Korea; 3 Research Center for Silver Science, Institute of Symbiotic Life-TECH, Yonsei University, Seoul, 03722 South Korea; 4 Division of Endocrinology and Metabolism, Department of Internal Medicine, Yonsei University College of Medicine, Seoul, 03722 South Korea

**Keywords:** Visceral fat area, Overweight, Fatty acids, Fatty acid desaturase, Fatty acid metabolism

## Abstract

**Background:**

Visceral fat accumulation in overweight status has been resulted in changes of fatty acid profiles. The fatty acids profiles can be altered by fatty acid desaturase; the activity of which is highly associated with obesity and other metabolic diseases. We hypothesized that fatty acid composition, desaturase activity, and accumulation of visceral fat are interrelated. Thus, the aim of this study was to investigate the association between increased visceral fat area and alterations in plasma fatty acid profile in overweight subjects with different amounts of visceral fat.

**Methods:**

Healthy overweight subjects (25.0 kg/m^2^ ≤ BMI < 30 kg/m^2^, *n*=232) were classified into lower (T1), middle (T2), and upper tertiles (T3) according to L4 visceral fat area (T1: <71.8 cm^2^, T2: 71.8 cm^2^–99.6 cm^2^, T3: >99.6 cm^2^).

**Results:**

The T3 group showed higher amounts of cis-10-heptadecenoic acid and activity of C16 Δ9-desaturase and C18 Δ9-desaturase and lower activity of Δ5-desaturase than the T1 group. Additionally, the T3 group showed higher amounts of saturated fatty acids, myristic acid, palmitic acid, stearic acid, monounsaturated fatty acids, palmitoleic acid, oleic acid, n-6 polyunsaturated fatty acids, linoleic acid, dihomo-γ-linolenic acid, arachidonic acid, n-3 PUFAs, and docosapentaenoic acid than the T1 and T2 groups.

**Conclusions:**

This study indicates that greater than a certain area (>99.6 cm^2^) of visceral fat is needed to observe altered levels of individual fatty acid species and desaturase activities. The results suggest that increased activity of C16 Δ9-desaturase and C18 Δ9-desaturase in parallel with decreased Δ5-desaturase activity may be a causative factor in disturbed fatty acid metabolism.

## Background

Obesity, particularly abdominal obesity, results from an increase in visceral fat. Visceral fat accumulation in overweight status has been suggested to lead to changes in the composition of lipids, lipoproteins [1], and fatty acids [2]. The fatty acid profile can be altered by diet as well as fatty acid desaturases [3]. These enzymes play an important role in regulating whole-body lipid composition [4, 5], and their activity is highly associated with obesity and other metabolic diseases [6]. For example, high Δ9-desaturase (D9D) activity has been associated with obesity, hypertriglyceridemia, metabolic syndrome, and increased risk of insulin resistance (IR) [6]; while, Δ5-desaturase (D5D) activity is negatively associated with the risk of metabolic syndrome and cardiovascular death [7–9]. Additionally, hepatic lipid composition changes caused by obesity are related to desaturase expression [10]. The results of a recent meta-analysis confirmed that the long-chain polyunsaturated fatty acid (PUFA) profile is altered in obesity; the differences observed in desaturase activity may be responsible for the disturbance of long-chain PUFA metabolism in overweight/obesity [11]. The study indicated high Δ6-desaturase (D6D) activity and low D5D activity in overweight and/or obese subjects. In detail, they observed low linoleic acid (C18:2, n-6) levels and high dihomo-γ-linolenic acid (C20:3, n-6) levels. Amount of dihomo-γ-linolenic acid (C20:3, n-6) in common foods is very low, therefore, the results mean that activity of D6D that contributes to convert linoleic acid (C18:2, n-6) to dihomo-γ-linolenic acid (C20:3, n-6) was increased in overweight and/or obese subjects. In addition, in those subjects, although their dihomo-γ-linolenic acid (C20:3, n-6) was high, the ratio of arachidonic acid (C20:4, n-6) to dihomo-γ-linolenic acid (C20:3, n-6) was low; that means the activity of D5D that converts dihomo-γ-linolenic acid (C20:3, n-6) to arachidonic acid (C20:4, n-6) was decreased [11]. Therefore, overweight and/or obese status, fatty acid composition, and desaturase activity are related to each other.

In this study, we hypothesized that fat distribution affects fatty acid composition and desaturase activity even in overweight individuals have similar fat mass; because fatty acid composition, desaturase activity, and accumulation of visceral fat are interrelated to some extent [12–15]. Moreover, the amount of visceral fat is important for assessing the impact on metabolic disease [16]. Therefore, we focused on the association between increased visceral fat area and alterations in the plasma fatty acid profile in overweight subjects.

## Methods

### Study subjects and design

Participants were recruited through advertisements in Seoul. Based on data from the Clinical Nutrition Laboratory, Yonsei University, overweight subjects [25.0 kg/m^2^ ≤ body mass index (BMI) <30 kg/m^2^] were referred to the department of Endocrinology, Yonsei University Hospital. They were interviewed and underwent blood and health tests; those who met the study criteria were then recommended to participate in this study. The participation criteria were age between 20 and 65 years; absence of pregnancy or breast-feeding; stable body weight (body-weight change < 1 kg over the 3 months before screening); no hypertension, type 2 diabetes, cardiovascular disease, or thyroid disease; and no use of any medication affecting body weight, lipid/fatty acid profiles, energy expenditure, or glucose control for the past 6 months. Additionally, only those who voluntarily consented to the program were included. Subjects were excluded if they drank more than 70 g of alcohol per week or had a prior history of Cushing disease, malignancy, or other liver diseases, including chronic viral hepatitis, autoimmune hepatitis, primary biliary cirrhosis, and drug-induced liver disease. Finally, subjects with a history of intentional weight loss in the last 6 months were excluded. In total, 232 healthy overweight (BMI between 25.0 and 30.0 kg/m^2^) individuals were enrolled and classified into lower (T1), middle (T2), and upper tertiles (T3) according to visceral fat area at L4 (T1: <71.8 cm^2^, T2: 71.8 cm^2^–99.6 cm^2^, T3: >99.6 cm^2^). Associations between visceral fat content and fatty acid profiles were observed cross-sectionally. We gave all subjects a careful explanation of the purpose of the study and received written consent prior to their participation. The protocol used in the study was approved by the Institutional Review Board of Yonsei University and Yonsei University Severance Hospital according to the Helsinki Declaration.

### Blood collection

Venous blood specimens were collected after an overnight fast of at least 12 h. The blood samples were collected in serum tubes and EDTA-treated whole-blood tubes (BD Vacutainer; Becton, Dickinson and Company, Franklin Lakes, NJ, USA). The samples were centrifuged to obtain plasma and serum from the whole blood and then stored at −80 °C.

### Anthropometric parameters and body composition measurements

Body weight (Inbody370; Biospace, Cheonan, Korea) and height (GL-150; G-tech International, Uijeongbu, Korea) were measured, and BMI (kg/m^2^) was calculated from these data. Waist circumference was assessed directly on the skin with a plastic measuring tape at the umbilical level. Systolic and diastolic blood pressure (BP) were measured with the participant in the supine position after a 20-min resting period. BP was assessed twice with an automatic BP monitor (FT-200S; Jawon Medical, Gyeongsan, Korea), and the average value of the two measurements was used.

Abdominal fat distribution data at L1 and L4 were obtained via computed tomography (CT) scanning using a GE Medical System HiSpeed Advantage® system (Milwaukee, WI, USA). The scanning parameters were a slice thickness of 1 mm at 200 mA and 120 kVp, with a 48-cm field of view. The body composition of the study subjects was measured with dual-energy X-ray absorptiometry (DEXA; Discovery A; Hologic, Bedford, MA, USA) to determine fat mass, lean body mass and fat percentage. The results were analyzed by volume integration software (APEX 4.0.2 [13.4.1]; Hologic, Bedford, MA, USA).

### Serum fasting glucose, serum insulin, and homeostatic model assessment (HOMA) of IR

Fasting glucose was assessed via the hexokinase method using Glucose Kits (Roche, Mannheim, Germany). Serum insulin was measured by an immunoradiometric assay using an Insulin Immunoradiometric Assay Kit (DIAsource, Louvain, Belgium). The resulting color reaction was monitored via a Hitachi 7600 system (Hitachi, Tokyo, Japan) for serum fasting glucose and an SR-300 system (Stratec, Birkenfeld, Germany) for insulin. IR was calculated from the HOMA using the following equation: HOMA-IR = [fasting insulin (μIU/mL) × fasting glucose (mg/dL)]/405.

### Serum fasting lipid profile and apolipoproteins

Fasting total cholesterol (TC) and triglyceride (TG) were measured through enzymatic assays using CHOL and TG Kits (Roche, Mannheim, Germany), respectively. Analysis of high-density lipoprotein (HDL-) cholesterol was conducted via selective inhibition using an HDL-C Plus Kit (Roche, Mannheim, Germany). The result of the color reaction was measured with a Hitachi 7600 autoanalyzer (Hitachi, Tokyo, Japan). Low-density lipoprotein (LDL-) cholesterol levels were calculated by the Friedewald formula: LDL-cholesterol = TC – [HDL-cholesterol + (TG/5)].

Serum fasting apolipoprotein B and apolipoprotein A-I were measured via immunoturbidimetric assays with Apolipoprotein B and Apolipoprotein A-I Kits (Roche, Mannheim, Germany), respectively. Turbidity was assessed using a Cobas Integra 800 autoanalyzer (Roche, Rotkreuz, Switzerland).

### Serum high-sensitivity C-reactive protein (hs-CRP)

The serum hs-CRP concentration was analyzed with CRP kits (Roche, Mannheim, Germany), and turbidity was monitored using a Hitachi 7600 autoanalyzer (Hitachi, Tokyo, Japan).

### Gas chromatography-mass spectrometry (GC-MS) analysis

The details regarding GC-MS analysis have been previously described [17]. Briefly, GC-MS analysis of metabolites in plasma was carried out using an Agilent Technologies 7890 N gas chromatograph coupled to an Agilent Technologies 5977A quadrupole mass selective spectrometer with a triple-axis detector (Agilent, Palo Alto, CA) operated in electron ionization mode at 70 eV with a mass scan range of m/z 50–800. Derivatized samples were separated on a VF-WAX column (Agilent Technologies, Middelburg, The Netherlands) with an oven temperature ramp from 50 °C to 230 °C. The carrier gas was helium set at constant flow mode (1.0 mL/min). The identification of each metabolite in the samples was confirmed by comparing their relative retention times and mass spectra with those of authentic standard compounds. The relative levels of metabolites were calculated by comparing their peak areas to that of the internal standard compound.

Fatty acid desaturase activities and elongase activities were obtained indirectly by calculating fatty acid ratios of products to precursors. The equations are as follows: C16 Δ9-desaturase = Palmitoleic acid/Palmitic acid; C18 Δ9-desaturase = Oleic acid/Stearic acid; Δ6-desaturase = γ-Linolenic acid/Linoleic acid; Elongase activity = Stearic acid/Palmitic acid.

### Statistical analysis

Statistical analysis was carried out in SPSS version 23.0 (IBM/SPSS, Chicago, IL, USA). The skewed variables were tested by logarithmic transformation. One-way analysis of variance (ANOVA) with a Bonferroni post hoc test was performed for continuous variables, and a chi-square test was applied to analyze nominal variables. A general linear model was used to adjust for the potential confounding factors, including age, sex, smoking, drinking, and BMI. Pearson’s correlation coefficient was analyzed to find relationships between variables and partial correlation analysis was performed to adjust for the confounding factors. A heat map was created via Multiple Experiment Viewer (MeV) version 4.9.0 (http://www.tm4.org/) to visualize the relationships between variables. The outcomes are stated as the mean ± standard error (SE). The results were considered statistically significant at *p* < 0.05.

## Results

### General characteristics of the study subjects

The general characteristics of 232 overweight subjects distributed over the tertiles of L4 visceral fat area are presented in Table 1. Of the three groups that we defined according to L4 visceral fat area (T1: <71.8 cm^2^, T2: 71.8 cm^2^–99.6 cm^2^, T3: >99.6 cm^2^), the T3 group was on average older and heavier than the others. After adjusting for age, sex, smoking, drinking, and BMI, the T2 and T3 groups showed higher fasting insulin, and a higher HOMA-IR index than the T1 group. After adjusting for confounding factors, the T3 group showed higher waist circumference, diastolic BP, and hs-CRP and lower HDL-cholesterol than did the T1 group as well as higher TG, TC, LDL-cholesterol, and apolipoprotein B than did the T1 and T2 groups. When each tertile was compared to the total subjects, there were no significant differences between the total subjects and the T2 group. The subjects in the T1 group were younger; had lower waist circumference, waist-to-hip ratio, diastolic BP, glucose, TG, total-cholesterol, LDL-cholesterol, apolipoprotein B, and hs-CRP; and had higher HDL-cholesterol than the total subjects. The subjects in the T3 group were older; showed increased waist circumference, waist-to-hip ratio, diastolic BP, TG, TC, LDL-cholesterol, apolipoprotein B, and hs-CRP; and showed decreased HDL-cholesterol compared to the total subjects.

**Table 1:** Clinical and biochemical characteristics of the subjects according to tertile of visceral fat area (VFA) at L4

	Total subjects (*n* = 232)	Lower tertile (T1, *n* = 77)	Middle tertile (T2, *n* = 78)	Upper tertile (T3, *n* = 77)	*p* ^*a*^	*p* ^*b*^	*p* ^*c*^
		VFA at L4 < 71.8 cm^2^	71.8 cm^2^ ≤ VFA at L4 ≤ 99.6 cm^2^	VFA at L4 > 99.6 cm^2^			
Age (year)	40.2 ± 0.68	34.6 ± 1.04^***^	40.7 ± 1.10	45.2 ± 1.10^***^	<0.001	–	–
Male/Female *n*, (%)	68 (29.3)/164 (70.7)	16 (20.8)/79.2 (79.2)	26 (33.3)/52 (66.7)	26 (33.8)/51 (66.2)	0.132	–	–
Cigarette smoker *n*, (%)	19 (8.2)	3 (3.90)	8 (10.3)	8 (10.4)	0.243	–	–
Alcohol drinker *n*, (%)	144 (62.1)	50 (64.9)	47 (60.3)	47 (61.0)	0.814	–	–
Weight (kg)	72.0 ± 0.59	71.4 ± 1.00	71.9 ± 1.08	72.7 ± 1.00	0.649	0.207	0.874
BMI (kg/m^2^)	26.9 ± 0.09	26.8 ± 0.17	26.7 ± 0.16	27.3 ± 0.16	0.040	0.007	–
Waist circumference (cm)	91.2 ± 0.37	89.2 ± 0.67^b,**^	91.5 ± 0.60^*a,b*^	92.9 ± 0.58^a,*^	<0.001	<0.001	0.003
Waist hip ratio	0.90 ± 0.00	0.88 ± 0.01^b,**^	0.90 ± 0.00^*a,b*^	0.92 ± 0.00^a,**^	<0.001	<0.001	0.001
Systolic BP (mmHg)	119.4 ± 0.87	116.7 ± 1.40	119.0 ± 1.69	122.5 ± 1.36	0.024	0.138	0.211
Diastolic BP (mmHg)	74.0 ± 0.64	71.2 ± 1.04^b,*^	73.8 ± 1.14^*a,b*^	77.0 ± 1.06^a,*^	0.001	0.016	0.021
Glucose (mg/dL)^*∮*^	87.1 ± 0.59	84.4 ± 0.95^*^	87.6 ± 1.03	89.2 ± 1.04	0.005	0.291	0.237
Insulin (μIU/dL)^*∮*^	13.5 ± 0.47	12.2 ± 0.53^*b*^	13.8 ± 1.06^*a*^	14.5 ± 0.77^*a*^	0.067	<0.001	<0.001
HOMA-IR^*∮*^	2.92 ± 0.11	2.56 ± 0.12^*b*^	3.02 ± 0.24^*a*^	3.19 ± 0.17^*a*^	0.012	<0.001	<0.001
Triglyceride (mg/dL)^*∮*^	127.7 ± 7.66	94.0 ± 4.73^b,**^	134.6 ± 19.2^*b*^	154.5 ± 10.6^a,**^	<0.001	<0.001	<0.001
Total cholesterol (mg/dL)^*∮*^	202.6 ± 2.67	190.9 ± 3.82^b,*^	196.6 ± 4.83^*b*^	220.3 ± 4.49^a,**^	<0.001	0.002	0.004
HDL-cholesterol (mg/dL)^*∮*^	54.1 ± 0.78	58.4 ± 1.30^a,**^	53.1 ± 1.31^*a,b*^	50.8 ± 1.32^b,*^	<0.001	0.006	0.008
LDL-cholesterol (mg/dL)^*∮*^	123.4 ± 2.24	113.7 ± 3.69^b,*^	117.2 ± 3.50^*b*^	139.8 ± 3.80^a,***^	<0.001	0.002	0.005
Apolipoprotein A-I (mg/dL)^*∮*^	152.1 ± 1.56	157.7 ± 2.84	150.4 ± 2.39	148.2 ± 2.79	0.029	0.148	0.183
Apolipoprotein B (mg/dL)^*∮*^	103.7 ± 1.88	90.9 ± 2.87^b,**^	99.4 ± 2.69^*b*^	120.9 ± 3.22^a,***^	<0.001	<0.001	<0.001
hs-CRP (mg/L)^*∮*^	1.27 ± 0.11	0.81 ± 0.12^b,**^	1.33 ± 0.20^*a,b*^	1.66 ± 0.22^a,**^	<0.001	<0.001	<0.001

Mean ± SE.^∮^tested by logarithmic transformation. *p* ^*a*^-values derived from a Chi-square test or One-way ANOVA for the nominal or continuous variables, respectively, among lower, middle, and upper tertile groups. *p* ^*b*^-values were adjusted for age, sex, smoking, and drinking. *p* ^*c*^-values were adjusted for age, sex, smoking, drinking, and BMI. All *p* < 0.05 marked with letters were derived from Bonferroni post hoc test at *p* ^*c*^-values; no significant differences are marked with the same letter and significant differences are marked with different letters. ^*^ *p* < 0.05, ^**^ *p* < 0.01, and ^***^ *p* < 0.001 derived from an independent *t*-test between the total subjects and each tertile group

### Visceral fat area and body composition measured by CT and DEXA

Table 2 shows visceral fat area measured using CT at L1 and L4 and body composition measured using DEXA. After adjusting for age, sex, smoking, drinking, and BMI, the T3 group showed the greatest visceral fat area at the L1 and L4 levels, whereas the T1 group showed a greater subcutaneous fat area at the L4 level than did the T3 group. At the L1 level, the T3 and T2 groups showed higher visceral/subcutaneous fat ratios (VSRs) than the T1 group, and at the L4 level, the T3 group showed the highest VSR. With respect to body composition measured using DEXA, the T1 and T3 groups showed higher total fat percentages than the T2 group, and the T3 group had a greater total fat mass than the T2 group (Table 2).

**Table 2:** Abdominal fat area and body composition

	Lower tertile (T1, *n* = 77)	Middle tertile (T2, *n* = 78)	Upper tertile (T3, *n* = 77)	*p* ^*a*^	*p* ^*b*^	*p* ^*c*^
	VFA at L4 < 71.8 cm^2^	71.8 cm^2^ ≤ VFA at L4 ≤ 99.6 cm^2^	VFA at L4 > 99.6 cm^2^			
CT evaluation (L1)						
Total fat area (cm^2^)	224.7 ± 5.16^*b*^	244.4 ± 5.37^*a*^	269.9 ± 6.13^*a*^	<0.001	<0.001	<0.001
Visceral fat area (cm^2^)	81.7 ± 2.64^*c*^	111.9 ± 3.58^*b*^	133.0 ± 4.83^*a*^	<0.001	<0.001	<0.001
Subcutaneous fat area (cm^2^)	143.0 ± 3.83	132.6 ± 4.04	136.9 ± 4.41	0.197	0.338	0.869
Visceral/subcutaneous fat ratio	0.60 ± 0.02^*b*^	0.93 ± 0.05^*a*^	1.07 ± 0.06^*a*^	<0.001	<0.001	<0.001
CT evaluation (L4)						
Total fat area (cm^2^)	289.6 ± 5.04^*b*^	294.4 ± 5.17^*b*^	325.5 ± 4.35^*a*^	<0.001	<0.001	<0.001
Visceral fat area (cm^2^)	56.7 ± 1.17^*c*^	86.6 ± 0.92^*b*^	121.3 ± 1.93^*a*^	<0.001	<0.001	<0.001
Subcutaneous fat area (cm^2^)	232.9 ± 4.79^*a*^	207.8 ± 5.06^*a,b*^	204.2 ± 4.79^*b*^	<0.001	0.140	0.005
Visceral/subcutaneous fat ratio	0.25 ± 0.01^*c*^	0.44 ± 0.01^*b*^	0.63 ± 0.02^*a*^	<0.001	<0.001	<0.001
DEXA evaluation						
Fat percentage (%)	30.8 ± 0.63^*a*^	30.0 ± 0.67^*b*^	30.6 ± 0.67^*a*^	0.667	0.001	0.009
Fat mass (g)	22,156.2 ± 404.0^*a,b*^	21,711.5 ± 439.9^*b*^	22,487.8 ± 447.7^*a*^	0.442	0.001	0.023
Lean body mass (g)	48,345.1 ± 1031.4	49,338.9 ± 1071.0	49,637.8 ± 1025.6	0.658	0.402	0.158

Mean ± SE. *p* ^*a*^-values derived from a One-way ANOVA. *p* ^*b*^-values were adjusted for age, sex, smoking, and drinking. *p* ^*c*^-values were adjusted for age, sex, smoking, drinking, and BMI. All *p* < 0.05 marked with letters were derived from Bonferroni post hoc test at *p* ^*c*^-values; no significant differences are marked with the same letter and significant differences are marked with different letters

### Plasma fatty acid profile according to L4 visceral fat area

Table 3 shows the plasma fatty acid profiles of the subjects according to L4 visceral fat area. After adjusting for age, sex, smoking, drinking, and BMI, the T3 group showed higher amounts of saturated fatty acids (SFAs), myristic acid (C14:0), palmitic acid (C16:0), stearic acid (C18:0), monounsaturated fatty acids (MUFAs), palmitoleic acid (C16:1, n-7), and oleic acid (C18:1, n-9) than the T1 and T2 groups. The T3 group also showed higher cis-10-heptadecenoic acid (C17:1, n-7) than the T1 group. Similarly, after adjusting for age, sex, smoking, drinking, and BMI, the T3 group showed higher n-6 PUFAs, linoleic acid (C18:2, n-6), dihomo-γ-linolenic acid (C20:3, n-6), and arachidonic acid (C20:4, n-6) than the T1 and T2 groups. Additionally, after adjusting for age, sex, smoking, drinking, and BMI, the T3 group showed the highest n-3 PUFA level and had higher docosapentaenoic acid (C22:5, n-3) than the T1 and T2 groups. Furthermore, the T3 group showed higher estimated activity levels of C16 Δ9-desaturase and C18 Δ9-desaturase and a lower activity of Δ5-desaturase than the T1 group (Table 3).

**Table 3:** GC-MS analysis of fatty acids in the subjects according to tertiles of visceral fat area (VFA) at L4

Relative peak area	Lower tertile (T1, *n* = 77)	Middle tertile (T2, *n* = 78)	Upper tertile (T3, *n* = 77)	*p* ^*a*^	*p* ^*b*^	*p* ^*c*^
	VFA at L4 < 71.8 cm^2^	71.8 cm^2^ ≤ VFA at L4 ≤ 99.6 cm^2^	VFA at L4 > 99.6 cm^2^			
Saturated fatty acids	6.200 ± 0.111^*b*^	6.350 ± 0.169^*b*^	7.086 ± 0.139^*a*^	<0.001	0.002	0.002
Lauric acid (C12:0)	0.023 ± 0.001	0.028 ± 0.002	0.031 ± 0.002	0.002	0.072	0.059
Myristic acid (C14:0)	0.200 ± 0.009^*b*^	0.230 ± 0.014^*b*^	0.289 ± 0.015^*a*^	<0.001	0.001	0.001
Pentadecylic acid (C15:0)	0.037 ± 0.001	0.041 ± 0.002	0.044 ± 0.001	0.003	0.147	0.149
Palmitic acid (C16:0)	4.074 ± 0.081^*b*^	4.128 ± 0.104^*b*^	4.613 ± 0.096^*a*^	<0.001	0.002	0.002
Stearic acid (C18:0)	1.739 ± 0.028^*b*^	1.799 ± 0.052^*b*^	1.969 ± 0.037^*a*^	<0.001	0.018	0.022
Arachidic acid (C20:0)	0.038 ± 0.001	0.038 ± 0.001	0.041 ± 0.001	0.060	0.194	0.262
Behenic acid (C22:0)	0.090 ± 0.003	0.087 ± 0.003	0.098 ± 0.003	0.017	0.049	0.091
Monounsaturated fatty acid	1.198 ± 0.029^*b*^	1.267 ± 0.047^*b*^	1.429 ± 0.036^*a*^	<0.001	0.003	0.003
Palmitoleic acid (C16:1, n-7)	0.139 ± 0.007^*b*^	0.149 ± 0.007^*b*^	0.183 ± 0.008^*a*^	<0.001	0.002	0.003
Cis-10-heptadecenoic acid (C17:1, n-7)	0.011 ± 0.000^*b*^	0.012 ± 0.001^*a,b*^	0.014 ± 0.000^*a*^	<0.001	0.022	0.025
Oleic acid (C18:1, n-9)	0.984 ± 0.022^*b*^	1.043 ± 0.038^*b*^	1.162 ± 0.029^*a*^	<0.001	0.004	0.004
Eicosenoic acid (C20:1, n-9)	0.009 ± 0.000	0.011 ± 0.001	0.011 ± 0.000	0.180	0.556	0.586
Erucic acid (C22:1, n-9)	0.009 ± 0.001	0.009 ± 0.001	0.009 ± 0.001	0.832	0.840	0.824
Nervonic acid (C24:1, n-9)	0.046 ± 0.001	0.044 ± 0.001	0.049 ± 0.002	0.099	0.197	0.297
Polyunsaturated fatty acids (n-6)	2.685 ± 0.052^*b*^	2.691 ± 0.059^*b*^	2.973 ± 0.049^*a*^	<0.001	0.004	0.003
Linoleic acid (C18:2, n-6)	1.980 ± 0.039^*b*^	2.001 ± 0.048^*b*^	2.205 ± 0.038^*a*^	<0.001	0.005	0.004
γ-linolenic acid (C18:3, n-6)	0.029 ± 0.002	0.030 ± 0.002	0.035 ± 0.002	0.104	0.438	0.343
Eicosadienoic acid (C20:2, n-6)	0.018 ± 0.001	0.021 ± 0.001	0.022 ± 0.001	0.017	0.251	0.212
Dihomo-γ-linolenic acid (C20:3, n-6)	0.095 ± 0.003^*b*^	0.101 ± 0.004^*b*^	0.118 ± 0.004^*a*^	<0.001	0.007	0.012
Arachidonic acid (C20:4, n-6)	0.532 ± 0.013^*b*^	0.510 ± 0.012^*b*^	0.558 ± 0.013^*a*^	0.030	0.040	0.038
Docosatetraenoic acid (C22:4, n-6)	0.030 ± 0.001	0.029 ± 0.001	0.034 ± 0.001	0.006	0.028	0.061
Polyunsaturated fatty acids (n-3)	0.357 ± 0.016^*b*^	0.415 ± 0.020^*b*^	0.499 ± 0.021^*a*^	<0.001	0.039	0.047
α-linolenic acid (C18:3, n-3)	0.079 ± 0.004	0.100 ± 0.009	0.113 ± 0.006	0.002	0.293	0.270
Eicosapentaenoic acid (C20:5, n-3)	0.080 ± 0.005	0.092 ± 0.006	0.113 ± 0.006	<0.001	0.393	0.494
Docosapentaenoic acid (C22:5, n-3)	0.036 ± 0.001^*b*^	0.040 ± 0.002^*b*^	0.049 ± 0.002^*a*^	<0.001	0.013	0.011
Docosahexaenoic acid (C22:6, n-3)	0.162 ± 0.009	0.183 ± 0.011	0.225 ± 0.014	0.001	0.094	0.113
Ratio n-6/n-3	8.434 ± 0.321	7.350 ± 0.284	6.587 ± 0.244	<0.001	0.149	0.191
C16 Δ9-desaturase^*a*^	0.033 ± 0.001^*b*^	0.035 ± 0.001^*a,b*^	0.039 ± 0.001^*a*^	0.002	0.021	0.029
C18 Δ9-desaturase^*b*^	0.565 ± 0.008^*b*^	0.577 ± 0.008^*a,b*^	0.589 ± 0.008^*a*^	0.102	0.047	0.036
Δ6-desaturase^*c*^	0.015 ± 0.001	0.015 ± 0.001	0.016 ± 0.001	0.579	0.815	0.813
Δ5-desaturase^*d*^	6.008 ± 0.217^*a*^	5.368 ± 0.163^*a,b*^	4.945 ± 0.143^*b*^	<0.001	0.025	0.036
Elongase^*e*^	0.432 ± 0.006	0.437 ± 0.006	0.430 ± 0.006	0.652	0.446	0.463

Mean ± SE. *p* ^*a*^-values derived from a One-way ANOVA. *p* ^*b*^-values were adjusted for age, sex, smoking, and drinking. *p* ^*c*^-values were adjusted for age, sex, smoking, drinking, and BMI. All *p* < 0.05 marked with letters were derived from Bonferroni post hoc test at *p* ^*c*^-values; no significant differences are marked with the same letter, and significant differences are marked with different letters. ^*a*^C16 Δ9-desaturase = Palmitoleic acid/Palmitic acid. ^*b*^C18 Δ9-desaturase = Oleic acid/Stearic acid. ^*c*^Δ6-desaturase = γ-Linolenic acid/Linoleic acid. ^*d*^Δ5-desaturase = Arachidonic acid/dihomo-γ-linolenic acid. ^*e*^Elongase activity = Stearic acid/Palmitic acid

### Correlation analysis of CT and DEXA parameters, major fatty acids, and fatty acid desaturases in all study participants

**Fig. 1:**
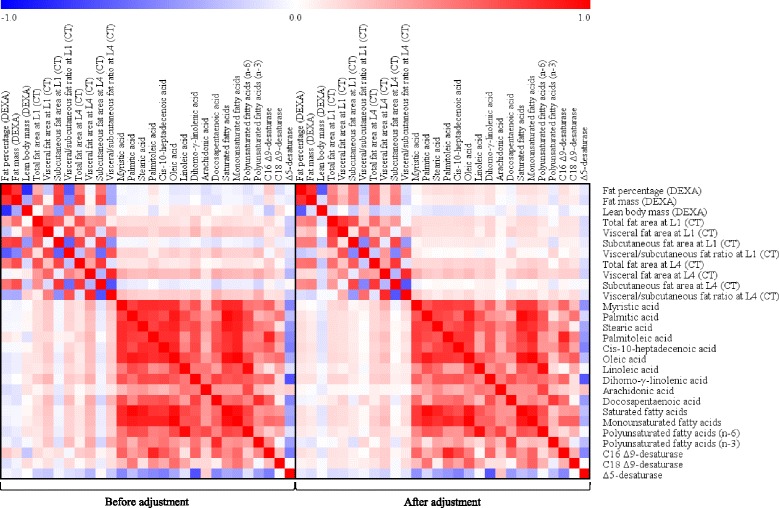
Correlation matrix of CT and DEXA parameters, major fatty acids, and fatty acids desaturases in all study participants before and after adjusting for confounding factors. Correlations were obtained by deriving Pearson’s correlation coefficient (left box). Age, sex, smoking, drinking, and BMI were adjusted (partial correlation, right box). *Red* indicates a positive correlation and *Blue* indicates a negative correlation

Based on the results of GC-MS analysis, fatty acids and fatty acid desaturases that showed significance after adjusting for confounding factors, including age, sex, smoking, drinking, and BMI, were included in a correlation analysis with CT and DEXA parameters. As shown in Fig. 1, before adjusting for confounding factors, VFA at L4 showed significant positive correlations with C16 Δ9-desaturase (*r* = 0.237, *p* < 0.001) and C18 Δ9-desaturase (*r* = 0.148, *p* = 0.024); and a significant negative correlation with Δ5-desaturase (*r* = −0.288, *p* < 0.001). In addition, VFA at L4 showed strong positive associations with all major fatty acids except for arachidonic acid (C20:4, n-6). These significant correlations remained after adjusting for the confounding factors. VFA at L1, before adjusting for confounding factors, showed a significant negative correlation with Δ5-desaturase (*r* = −0.217, *p* = 0.001) and significant positive correlations with all major fatty acids; but no significant correlation with C16 Δ9-desaturase or C18 Δ9-desaturase was found. After adjustment, myristic acid (C14:0), oleic acid (C18:1, n-9), dihomo-γ-linolenic acid (C20:3, n-6), docosapentaenoic acid (C22:5, n-3), saturated fatty acids, and monounsaturated fatty acids showed significant positive correlations with VFA at L1; and the significant correlations with regard to the fatty acid desaturases remained the same as prior to the adjustment.

## Discussion

In contrast to subcutaneous fat, visceral adipose tissues are metabolically active, sensitive to lipolysis, and insulin-resistant [15]. Because of these characteristics, visceral obesity causes several metabolic dysregulations including altered lipid profiles (e.g. high levels of TG and apolipoprotein B and low levels of HDL-cholesterol), altered glucose homeostasis (e.g. high levels of glucose and insulin and high HOMA-IR indices), and deteriorative inflammation status (e.g. high hs-CRP) [18, 19].

Due to the hyperlipolytic properties of the visceral adiposity, excess visceral fat releases large amount of fatty acids; hence, influx of fatty acids from visceral adipose tissues to the liver via the portal vein is increased [20–23]. In addition, Nielsen et al. [23] demonstrated that fatty acid delivery from visceral fat into hepatocytes increases with increasing visceral fat mass. This leads to increased fatty acid availability in hepatocytes, thereby stimulating hepatic synthesis and secretion of TG via its incorporation into TG-rich lipoproteins such as very low-density lipoproteins (VLDLs) [21, 23, 24]; finally, circulating TG increase according to visceral fat accumulation. Moreover, both the concentrations of portal vein fatty acids and the levels of systemic circulating fatty acids showed significant and positive relationships with visceral adipose tissues [23]. Indeed, in the present study, the T3 group showed increased total circulating fatty acids (SFAs, MUFAs, and PUFAs), TG, and apolipoprotein B, a major component of VLDL, compared to the T1 and T2 groups; again, these results mean that TG was synthesized in the liver from the fatty acids released from the large amount of visceral adipose tissue in the T3 group.

With regards to circulating fatty acid composition, the present study indicates that overweight subjects in the T3 group (visceral fat area at L4 >99.6 cm^2^) showed altered levels of individual fatty acids, including myristic acid (C14:0), palmitic acid (C16:0), stearic acid (C18:0), palmitoleic acid (C16:1, n-7), oleic acid (C18:1, n-9), cis-10-heptadecenoic acid (C17:1, n-7), linoleic acid (C18:2, n-6), dihomo-γ-linolenic acid (C20:3, n-6), arachidonic acid (C20:4, n-6), and docosapentaenoic acid (C22:5, n-3), and altered estimated activities of C16 Δ9-desaturase, C18 Δ9-desaturase, and Δ5-desaturase after adjusting for age, sex, smoking, drinking, and BMI. However, there were no significant differences in individual fatty acid levels or desaturase activity between the T1 and T2 groups, although the T2 group had higher serum insulin and HOMA-IR index than the T1 group. These results suggest that greater than a certain area (>99.6 cm^2^) of visceral fat is needed to observe altered levels of individual fatty acid species and desaturase activity. Indeed, according to our correlation analysis, a larger accumulation of visceral fat area at L4 was associated with higher activities of C16 Δ9-desaturase and C18 Δ9-desaturase and lower activity of Δ5-desaturase; since fatty acid desaturases affect fatty acid profile [3], the differences in fatty acid profiles across the tertile groups can be elucidated by the correlations between visceral fat area and fatty acid desaturases.

In this study, the amount of total SFAs in plasma was significantly higher in the T3 group than those in the T1 and T2 groups; these SFAs include palmitic acid (C16:0) and stearic acid (C18:0), which are related to the incidence of type 2 diabetes [25, 26]. Additionally, total SFAs may increase cardiovascular disease risk by increasing levels of LDL-cholesterol and TC [27]. Indeed, the T3 group of this study showed higher TC and LDL-cholesterol than the T1 and T2 groups. Similarly, the level of total MUFAs, particularly plasma oleic acid (C18:1, n-9) and palmitoleic acid (C16:1, n-7), increased in the T3 group. We also found that overweight individuals in the T3 group had increased stearic acid (C18:0), oleic acid (C18:1, n-9), and C18 Δ9-desaturase. This might be explained by oversupply of fatty acids from excess visceral fat and altered activity of fatty acid desaturase according to visceral fat area.

Overweight individuals in the T3 group also had increased palmitic acid (C16:0), palmitoleic acid (C16:1, n-7), and C16 Δ9-desaturase. The serum concentration of palmitoleic acid (C16:1, n-7) mostly reflects de novo hepatic fatty acid synthesis from palmitic acid (16:0) by C16 Δ9-desaturase [28–30]; thus, high levels of palmitoleic acid (C16:1, n-7) in the T3 group indicate enhanced activity of C16 Δ9-desaturase with increasing visceral fat area. High levels of this particular fatty acid have been associated with an increased risk of cardiovascular disease as it has been positively associated with metabolic syndrome [7], including hypertriglyceridemia [31] and abdominal obesity [32]. Indeed, the T3 group in this study showed higher serum TG, waist circumference, and waist-to-hip ratio than the T1 group. Additionally, mice supplemented with palmitoleic acid (C16:1, n-7) presented higher fat deposition, hepatic steatosis, and increased hepatic expression of sterol regulatory element-binding protein 1c and fatty acid synthase, demonstrating the pro-lipogenic effect of this MUFA [33]. Therefore, high levels of palmitoleic acid (C16:1, n-7) via enhanced C16 Δ9-desaturase activity in overweight subjects with high visceral fat area may exacerbate fat accumulation.

The levels of total n-6 PUFAs, particularly linoleic acid (C18:2 n-6), dihomo-γ-linolenic acid (C20:3, n-6), and arachidonic acid (C20:4, n-6), were higher in the T3 group than those in the T1 and T2 groups. The n-6 PUFAs are thought to promote adipogenesis and increase the expression of lipogenic genes [34, 35]. The dihomo-γ-linolenic acid (C20:3, n-6) content of common foods is very low. Additionally, there was no significant difference in the level of Δ6-desaturase across the tertile groups. Thus, the increased dihomo-γ-linolenic acid (C20:3, n-6) content cannot be explained by a high dietary intake of dihomo-γ-linolenic acid (C20:3, n-6) or Δ6-desaturase but rather could indicate reduced Δ5-desaturase activity in overweight subjects with a high visceral fat area. Therefore, the limited rate of conversion of dihomo-γ-linolenic acid (C20:3, n-6) to arachidonic acid (C20:4, n-6) may also contribute to the accumulation of dihomo-γ-linolenic acid (C20:3, n-6) among the plasma lipids, which predicts an increased risk of metabolic syndrome and type 2 diabetes [2, 36, 37]. In contrast to n-6 PUFAs, this study showed only slight differences in the levels of n-3 PUFAs, specifically docosapentaenoic acid (C22:5, n-3), which was higher in the T3 group than that in the T1 and T2 groups.

In addition to altered fatty acid desaturase activity, the altered fatty acid levels and metabolic dysregulations observed in this study can also be explained by dysfunction of visceral adipose tissues. Adipose tissue dysfunction, which is involved in atherosclerotic vascular diseases and type 2 diabetes development, is a state of hypersecretion of pro-atherogenic, pro-inflammatory, and pro-diabetic adipocytokines [38]. Similarly, such characteristics were found in the T3 group of this study: atherogenic lipid profiles (high TG, TC, LDL-cholesterol, and apolipoprotein B and low HDL-cholesterol), inflammation (high hs-CRP), and risk of IR (high insulin and HOMA-IR). These features are similar to those that can be observed in individuals with excess visceral fat area [18, 19]. Therefore, adipose tissue dysfunction might have existed in the T3 group because of large visceral fat accumulation.

In summary, we found that excess visceral fat (greater than 99.6 cm^2^ in case of this study) may be associated with changes in circulating fatty acid composition, altered activities of fatty acid desaturases that disturb plasma fatty acid metabolism, and adipose tissue dysfunction. As discussed so far, such changes are related to cardiovascular diseases and/or type 2 diabetes; thus, visceral fat area should be observed to prevent these diseases.

The present study has several limitations. First, we only analyzed overweight Korean subjects. Body structure and habitual diets can differ substantially between races and countries. Second, this study is observational; thus, the causal relationship between fatty acid pattern and visceral fat area in overweight subjects was not proven. Third, we did not analyze fatty acids from each lipid fraction but rather from total lipids. Despite these limitations, the main finding of this study is that the different fatty acid composition in each overweight individual is due to different desaturase activities with increasing visceral fat area. Therefore, the results of the present study support the assumption that increased activities of C16 Δ9-desaturase and C18 Δ9-desaturase in parallel with decreased Δ5-desaturase activity may be a causative factor in disturbed fatty acid metabolism in overweight subjects with a high visceral fat area, particularly with the SFAs, MUFAs, and n-6 PUFA series.

Further high-quality studies including polymorphism analysis of fatty acid desaturases are required for a better understanding of the potential role of SFAs, MUFAs, and n-6 and n-3 PUFAs in the development and consequences of overweight/obesity with high visceral fat.

## Conclusion

Our results suggest that increased visceral fat area causes alterations in individual fatty acid levels and that altered desaturase activity due to visceral fat accumulation affects the composition of circulating fatty acids for the worse. These data support the assumption that increased activities of C16 Δ9-desaturase and C18 Δ9-desaturase in parallel with decreased Δ5-desaturase activity may result in disturbed fatty acid metabolism in overweight subjects with a high visceral fat area, particularly with the SFAs, MUFAs, and n-6 PUFA series. Additionally, atherogenic traits are worsened and adipose tissue dysfunction may occur in overweight subjects with large visceral fat accumulation. Therefore, individuals with high visceral fat area should focus on treating and preventing cardiovascular diseases.

## Abbreviations

ANOVA: One-way analysis of variance
BMI: Body mass index
BP: Blood pressure
CT: Computed tomography
DEXA: Dual-energy X-ray absorptiometry
GC-MS: Gas chromatography-mass spectrometry
HDL: High-density lipoprotein
HOMA: Homeostatic model assessment
hs-CRP: High-sensitivity C-reactive protein
IR: Insulin resistance
LDL: Low-density lipoprotein
MUFA: Monounsaturated fatty acid
PUFA: Polyunsaturated fatty acid
SE: Mean ± standard error
SFA: Saturated fatty acid
TC: Total cholesterol
TG: Triglyceride
VLDL: Very low-density lipoprotein
VSR: Visceral/subcutaneous fat ratio
